# Role of Mitochondrial Nucleic Acid Sensing Pathways in Health and Patho-Physiology

**DOI:** 10.3389/fcell.2022.796066

**Published:** 2022-02-11

**Authors:** Arpita Chowdhury, Steffen Witte, Abhishek Aich

**Affiliations:** ^1^ Department of Cellular Biochemistry, University Medical Center, Göttingen, Germany; ^2^ Cluster of Excellence “Multiscale Bioimaging, from Molecular Machines to Networks of Excitable Cells” (MBExC), University of Göttingen, Göttingen, Germany

**Keywords:** mitochondrion, disease, innate immunity, signaling, mitochondrial—nuclear exchange

## Abstract

Mitochondria, in symbiosis with the host cell, carry out a wide variety of functions from generating energy, regulating the metabolic processes, cell death to inflammation. The most prominent function of mitochondria relies on the oxidative phosphorylation (OXPHOS) system. OXPHOS heavily influences the mitochondrial-nuclear communication through a plethora of interconnected signaling pathways. Additionally, owing to the bacterial ancestry, mitochondria also harbor a large number of Damage Associated Molecular Patterns (DAMPs). These molecules relay the information about the state of the mitochondrial health and dysfunction to the innate immune system. Consequently, depending on the intracellular or extracellular nature of detection, different inflammatory pathways are elicited. One group of DAMPs, the mitochondrial nucleic acids, hijack the antiviral DNA or RNA sensing mechanisms such as the cGAS/STING and RIG-1/MAVS pathways. A pro-inflammatory response is invoked by these signals predominantly through type I interferon (T1-IFN) cytokines. This affects a wide range of organ systems which exhibit clinical presentations of auto-immune disorders. Interestingly, tumor cells too, have devised ingenious ways to use the mitochondrial DNA mediated cGAS-STING-IRF3 response to promote neoplastic transformations and develop tumor micro-environments. Thus, mitochondrial nucleic acid-sensing pathways are fundamental in understanding the source and nature of disease initiation and development. Apart from the pathological interest, recent studies also attempt to delineate the structural considerations for the release of nucleic acids across the mitochondrial membranes. Hence, this review presents a comprehensive overview of the different aspects of mitochondrial nucleic acid-sensing. It attempts to summarize the nature of the molecular patterns involved, their release and recognition in the cytoplasm and signaling. Finally, a major emphasis is given to elaborate the resulting patho-physiologies.

## Introduction

Mitochondria play an essential role in generating cellular energy and contain the oxidative phosphorylation (OXPHOS) system. Primary mitochondrial disorders are a complex group of metabolic impairments caused by flaws or deficiencies in one or more components of the OXPHOS. All these defects manifest in mitochondrial dysfunction which is sensed and communicated through a plethora of mitochondrial-nuclear signaling pathways (Mottis et al., 2019). However, in recent years, interferon-dependent innate immune responses have been shown to affect OXPHOS machinery and vice versa (Kiritsy et al., 2020; Buang et al., 2021). Defects in OXPHOS metabolism also result in the trigger of mitochondrial nucleic acid sensing pathways (Lei et al., 2021; Sprenger et al., 2021). Thus, to unravel this complex interaction, it is necessary to decipher how mitochondria communicate with the immune system.

## Foreign Nature of Mitochondria

The eukaryotic way of life is principally maintained by energy-transducing organelles, the mitochondria. An endosymbiotic event around two billion years ago led to the acquisition of this organelle. (Gray et al., 1999; Osteryoung and Nunnari, 2003). According to the widely accepted theory, a host cell engulfed an α-proteobacterium *via* endocytosis, which led to a double-membrane-bound organelle harboring its independent genetic material. Thus the host cell acquired the ability to couple catabolism of carbon fuels to ATP synthesis through OXPHOS. In course of evolution, functional redundancy led to the loss of the original proteobacterial genetic material. The rest which was essential was transferred to the nuclear genome (nDNA) of the host cell (Gray et al., 1999). In modern-day mammals, around 1,200 mitochondrial proteins are encoded by the nDNA (Pagliarini et al., 2008; Rath et al., 2020). These proteins are translated on the cytosolic ribosomes and subsequently imported into mitochondria through a dedicated mitochondrial protein import system (Dudek et al., 2013). However, mitochondria still retains a small fraction of the original proteobacterial genome within its matrix—mitochondrial DNA (mtDNA). In vertebrates, mtDNA contains intronless, polycistronic genes that encode only 13 mitochondrial proteins, 22 transfer RNAs and two ribosomal RNAs (Shadel and Clayton, 1997).

### Mitochondrial Organization and Dynamics

Like other organelles, mitochondria are maintained within the cell where they undergo biogenesis and turnover. They are also distributed among daughter cells following mitosis. Thus, the dynamic nature of mitochondria—fission, fusion and distribution—is responsible for its function and inheritance. Among the mitochondria in each cell, there are intricate networks of threads and smaller fragmented bodies depending on its health and function. Smaller mitochondrial organelles are generated when mitochondria divide, permitting efficient movement, organisation around the cell and inheritance. Mitochondrial fusion ensures that functionally and structurally homogeneous networks are formed by mixing material between organelles. Consequently, mitochondrial dynamics govern many aspects of the network, including organelle turnover, metabolism, cell stress and disease (Kraus et al., 2021).

**FIGURE 1:**
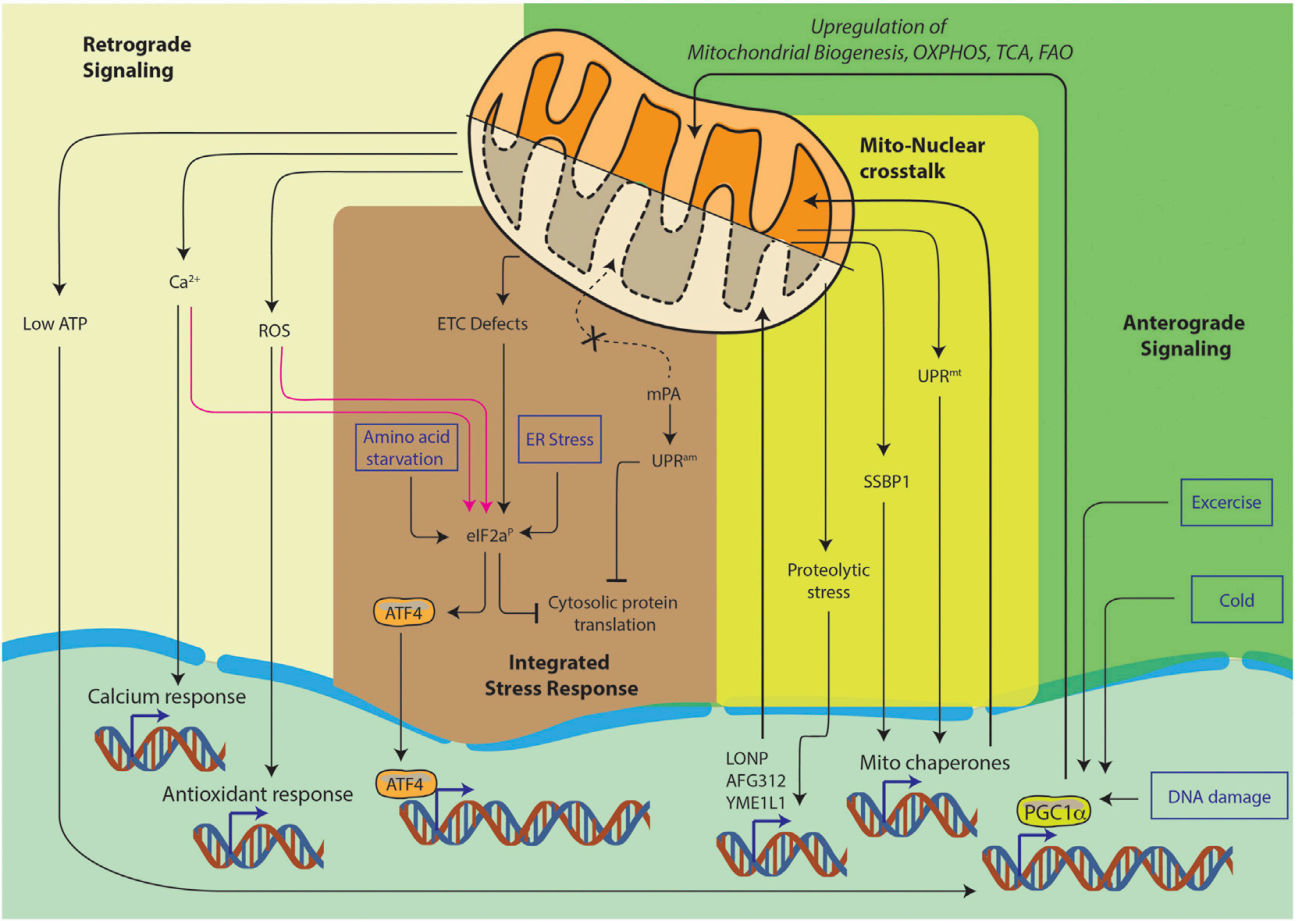
Pathways of nucleo-mitochondrial communication. Nucleus-to-mitochondrion signals make up anterograde signaling. Signals from the mitochondria constitute the retrograde signaling. The integrated stress response is a general cellular stress pathway which can be triggered in ER, mitochondria or cytosol. Lastly, mito-nuclear crosstalk is the ability to orchestrate bidirectional feedback responses, they usually originate in mitochondria and result in nuclear responses.

Furthermore, mitochondrial state also contributes to continuous nucleo-mitochondrial communication, such as the retrograde signaling, anterograde signaling, integrated stress response and mito-nuclear crosstalk (Figure 1). It is the complex signaling cascade that involves nuclear genes encoding transcription factors for mitochondrial maintenance. This directly affects OXPHOS structural heterogeneity and metabolic plasticity (Ryan and Hoogenraad, 2007). Furthermore, during stress conditions such as starvation or cell growth, contact sites among organelles can spatially regulate lipid synthesis, protein turnover and molecular trafficking. Moreover, the mitochondria associated membranes (MAMs), which are contact sites between the endoplasmic reticulum (ER) and the mitochondria, are responsible for the regulation of ROS generation, calcium homeostasis and autophagy (Quirós et al., 2016). Thus, to maintain the balance between cellular and mitochondrial health, several quality control mechanisms have evolved. Notably, the most studied ones are autophagy, mitophagy, translation attenuation processes and mitochondrial unfolded protein response (mtUPR) (Lechuga-Vieco et al., 2021). In recent years, a new paradigm of mitochondrial stress signaling is being uncovered. In this review, we would like to highlight this equally important pathway of mitochondrial nucleic acid sensing induced stress signaling.

### Mitochondrial Derived Damage Associated Molecular Patterns

**FIGURE 2:**
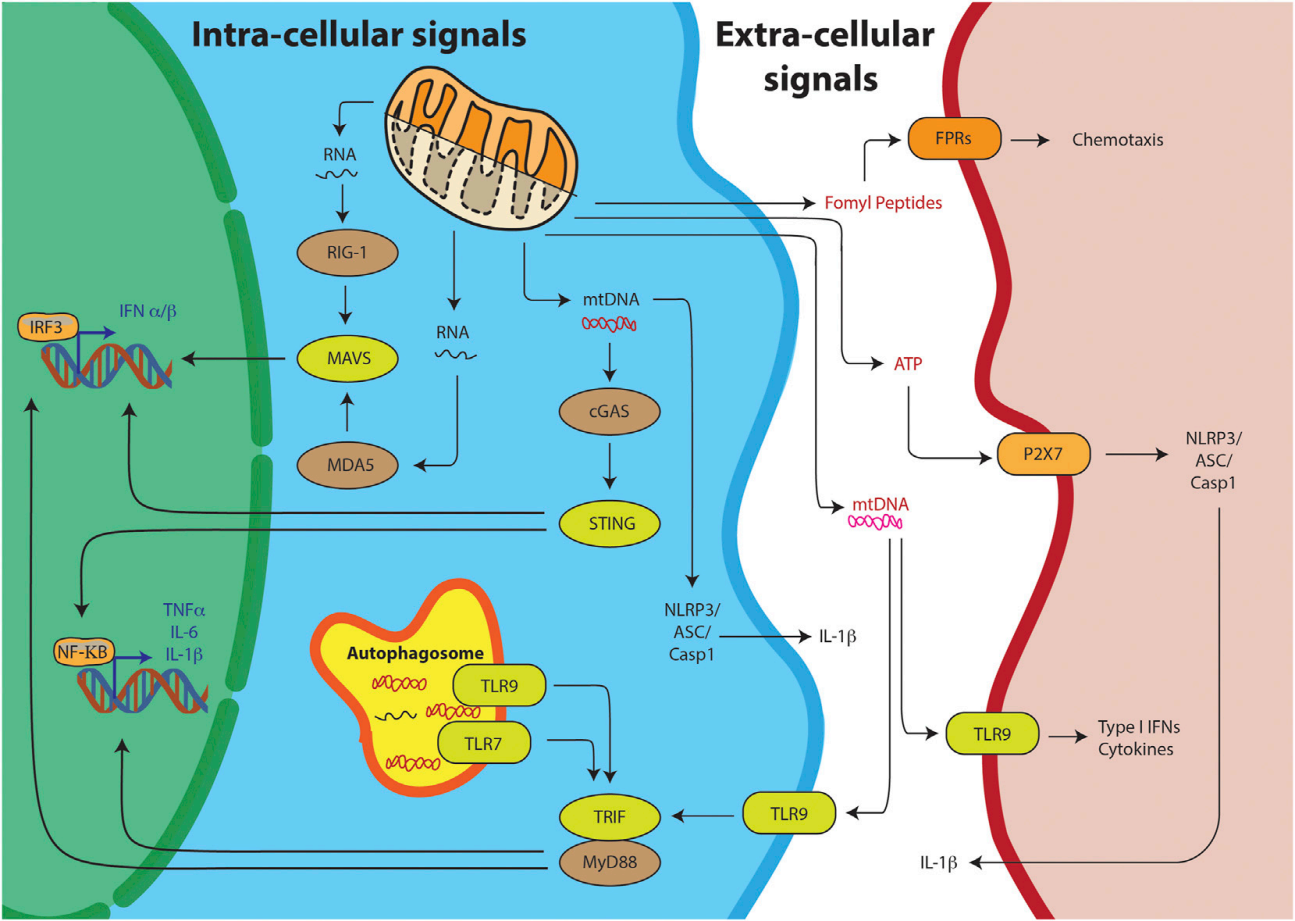
Mitochondrial DAMPs can trigger the innate immunity through various pathways. Intra-cellular signals include the sensing of the mitochondrial nucleic acids, mtRNA or mtDNA, resulting into IRF3 or NFKB mediated T1-IFN response. Alternatively, TLR receptors on the autophagosome can sense mitochondrial nucleic acids and through TRIF/MyD88 stimulate the T1-IFN signaling. Free mtDNA can also stimulate the inflammasome for IL-1B production. DAMPs can also stimulate their respective receptors on other cells through extra-cellular signaling.

Innate immunity is body’s first line of defense against pathogens and other invaders. It relies on sensing of two kinds of stimuli, Pathogen Associated Molecular Patterns (PAMPs) or Damage Associated Molecular Patterns (DAMPs) (Zindel and Kubes, 2019). PAMPs are foreign, pathogen-exclusive molecules meant to alert the immune system of an invader it needs to clear. DAMPs, on the other hand, are host produced molecules—usually sequestered away from the surveillance of the immune system in cellular compartments. But upon stress or cellular damage, these are released from their compartments and sensed as foreign, eliciting a similar innate immune response one would see against pathogens. The sensing of these molecules could be through intrinsic pathway or extrinsic pathway. In the intrinsic pathway they are sensed in the cytoplasm of the cells undergoing the damage, while in the extrinsic pathway the sensing occurs outside in the plasma by other cells such as the dendritic cells or monocytes (Figure 2). The chemical properties of the DAMPs decide its ability to trigger either of the pathways. Thus, it is important to look at some of the key mitochondria derived DAMPs (mtDs) and their mode of action.

Adenosine triphosphate (ATP) is the most prominent product of mitochondria, associated as the currency of energy in the cell. It is synthesized in the matrix of the mitochondria by ATP synthase coupled to the OXPHOS. Depending upon the demand it is translocated to the cytoplasm of the cell through the adenine nucleotide translocator (Ruprecht et al., 2019). Thus, under normal heathy physiology, ATP mostly stays intracellular. ATP exit the cells upon cellular damage, exocytosis and through ATP release channels (Taruno, 2018). Extracellular ATP associated with stress or damage is mostly pro-inflammatory (Faas et al., 2017). Through the activation of P2X ligand-gated ion channels and P2Y G protein-coupled receptors, extracellular ATP is able to act on virtually all subsets of immune cells (Cekic and Linden, 2016).

Another mtDs, succinate, is generated in the in mitochondria during energy metabolism *via* the tricarboxylic acid cycle (TCA). It may be released to the extracellular space through plasma membrane transporters of the SLC13 family (Willmes and Birkenfeld, 2013). Here, it is sensed by G protein-coupled receptors, GPR91/SUCNR1—which are expressed across a wide variety of tissues (Gilissen et al., 2016). Succinate has dual roles during inflammatory responses. It has either a pro- or anti-inflammatory role depending on the cellular context (Grimolizzi and Arranz, 2018). Studies on uncoupling protein 1 (UCP1) using the UCP1KO mice, show that UCP1-succinate–SUCNR1 axis is crucial for liver immune cell infiltration and pathology (Mills et al., 2021). On the other hand, it has been shown to hyperpolarize macrophages towards the M2 phenotype (Trauelsen et al., 2021).

The next mtDs, cardiolipin (CL), is a phospholipid which happens to be an important component of the inner mitochondrial membrane (IMM). Upon apoptotic signals, cellular infections and inflammatory diseases, it is translocated to the outer mitochondrial membrane (OMM) (Pizzuto and Pelegrin, 2020). Mitochondrial function and the inflammatory response to translocated cardiolipin depend on its saturation and oxidation status. Similar to succinate, it can have both pro- or anti-inflammatory roles. In addition to its direct sensing by CD1d on T-cells (Dieudé et al., 2011) and NLRP3 inflammasome (Iyer et al., 2013), it can also promote inflammation by blocking IL-10 production as shown in mice infected with *Klebsiella pneumoniae* (Chakraborty et al., 2017).

Another archaic remnant in the mitochondria is the process of N-formylation. The mitochondria still use N-formyl-methionyl-tRNA as an initiator of protein synthesis (Ayyub and Varshney, 2019). This process is also observed in bacteria and chloroplasts. Thus, damaged and dying mitochondria secrete N-formyl peptides which are picked up as chemotactic tails by polymorphonuclear cells (Wenceslau et al., 2014). These are recognized by FPR1 receptors (He and Ye, 2017). The outcome of FPR1 receptor engagement depends on the pathogen and disease. It either acts pro- or anti-inflammatory. This contrast can be seen in two different bacterial infections. In case of *Escherichia coli and Listeria monocytogenes*, FPR1 causes chemotactic recruitment of neutrophils, whereas contrarily it helps in the dissemination of *Yersinia pestis* (Vacchelli et al., 2020)*.*

Mitochondrial transcription factor A (TFAM) is another mtDs, which is a protein that binds nonspecifically in large number of copies to the mtDNA. It is responsible for the spatial organization and biogenesis of the same. Upon mitochondrial damage and dysfunction, extramitochondrial protein localization of TFAM increases significantly. Extracellular TFAM inevitably elicits a pro-inflammatory response (Little et al., 2014). Injection of TFAM in rat brains have shown that different cells types elicit an upregulation of inflammatory mediators such as monocyte chemotactic protein (MCP)-1, IL-1β, IL-6, tumor necrosis factor (TNF)-α (Schindler et al., 2018). It serves in promoting plasmacytoid Dendritic Cell responses to mtDNA through engagement of Toll like receptor (TLR) nine receptors (Julian et al., 2012).

The last group of mtDs, which happen to be the focus of this review, are mitochondrial nucleic acids. mtDNA, a double stranded circular molecule, is often represented as a plasmid structure. But it is always packed as densely compacted nucleoprotein structures called nucleoids. Typically, a nucleoid contains one to two mtDNA molecules in mammals (Kukat et al., 2011). Since the volume of the purified mtDNA significantly exceeds that of the mitochondria itself, a considerable compaction and organization with the help of more than 50 different proteins are required for this structure to be packed in the matrix (Bogenhagen et al., 2003). Apart from this, specific bodies called “mitochondrial RNA granules” were found in mitochondrial matrix. These are complexes of RNase P with newly synthesized mtRNA (Jourdain et al., 2016; Xavier and Martinou, 2021). Thus, a radial organization of the genetic material is hypothesized, with the nucleoid at the core, surrounded by ring of RNA granules which are further surrounded by a cloud of mRNA being translated. Since, both mitochondrial nucleic acids are well sequestered in the matrix of the mitochondria, these escape the self-nonself discrimination of both the innate and adaptive immunity arms. The detailed mechanism of release and detection of both of these moieties is discussed in the next sections. Furthermore, they are associated with several “auto-immune” diseases affecting a multitude of organ systems, thus demanding a comprehensive look as well.

## Mitochondria: The Hidden Player in Innate Immune Response

Innate immunity is an evolutionarily conserved host defense mechanism. Mammals possess innate immune defenses in nearly every tissue, including the skin and mucosal surfaces of the respiratory and digestive tracts. In response to tissue damage, heat shock, infections, genotoxic or carcinogenic stress, the hematopoietic myeloid and lymphoid cells can trigger and further exert innate defense mechanisms. This is primarily mediated by the release of endogenous molecules such as uric acid, ATP, pathogenic molecules (DNA, RNA, proteins), *N*-formyl peptides (NFPs), heparan sulfate etc. during the above-mentioned events. These molecules further activate the pattern recognition receptors (PRRs) on innate immune cells (Brennan et al., 2015) (Takeuchi and Akira, 2009). In the event of viral infections, certain products of viral infections like viral proteins and nucleic acids, which are also known as pathogen associated molecular patterns (PAMPs) are sensed by PRRs as non-self to elicit antiviral innate immune response. This is primarily directed through type I and type III interferons (IFN) (Mesev et al., 2019). The identification of Mitochondrial antiviral-signaling protein (MAVS) not only changed the conception of innate immune responses by viral infections, but also implicated a new role of mitochondria in innate immunity (Seth et al., 2005). MAVS, which is a 540-amino acid long protein, primarily localizes on the outer mitochondrial membrane (Meylan et al., 2005). Moreover, it has also been detected on mitochondrial associated endoplasmic reticulum membranes and peroxisomes (Horner et al., 2011; Bender et al., 2015). The role of MAVS as a key adaptor protein in eliciting and promoting signal transduction against RNA viruses, paved the way towards a deeper understanding of viral RNA sensing and antiviral responses. In the next sections we will discuss more in detail about the mechanisms of viral RNA and DNA sensing is association with MAVS expression and signaling function.

### RNA Sensors in the Cytosol

**FIGURE 3:**
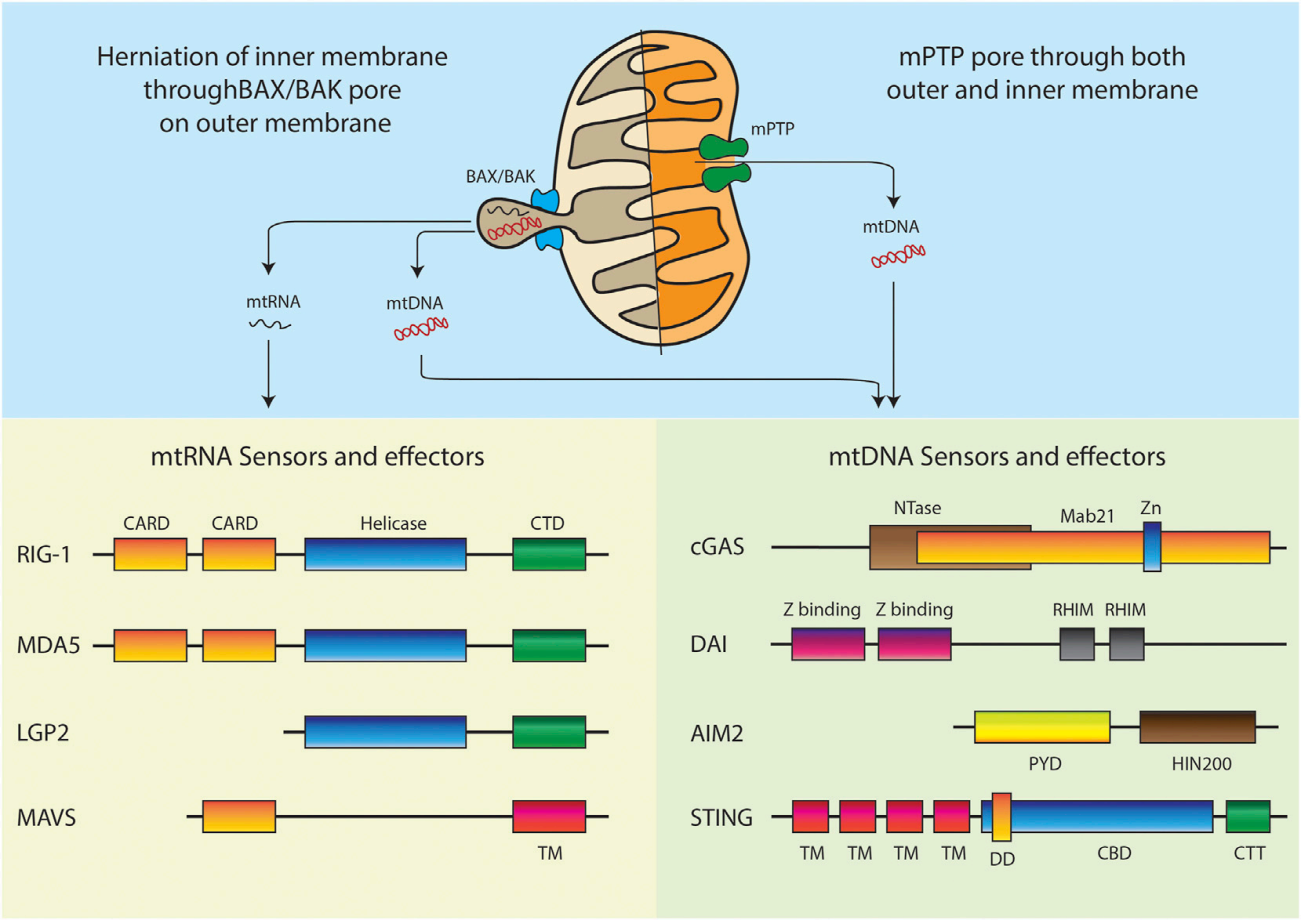
Models of mitochondrial nucleic acids release and respective intracellular sensors and effectors. Domain abbreviations: Caspase activation and recruitment domains (CARD), C-terminal regulatory domain (CTD), Transmembrane (TM), Nucleotidyltransferase (NTase), Zinc ribbon insertion (Zn), Receptor-interacting protein (RIP) Homotypic interaction motifs (RHIM), Pyrin domain (PYD), Hematopoietic interferon-inducible nuclear protein with 200-amino-acid repeats (HIN200), Dimerization domain (DD), Cyclic dinucleotide (CDN) binding domain (CBD), C-terminal Tail (CTT).

RIG-I-like receptors (RLRs) are a family of cytosolic pattern recognition receptors that play a pivotal role in detecting and distinguishing cytosolic viral RNA from cellular RNAs and activating downstream signaling events to initiate antiviral innate immune responses. This entire pathway is impelled by the interaction of RLRs with MAVS. MAVS consist of three domains: an N-terminal caspase recruitment domain (CARD), a middle proline-rich region, and a C-terminal transmembrane (TM) domain (Figure 3). While the RLR family primarily consists of three members: Retinoic acid-inducible gene I (RIG-I), Melanoma differentiation-associated gene 5 (MDA5), and Laboratory of genetics and physiology 2 (LGP2). All the three receptors of this family have a DExD/H box RNA helicase domain with ATPase activity and a carboxy-terminal domain (CTD). Binding of RNA requires both these domains. Additionally, the CTD of RIG-I and LGP2 has been shown to act as a repressor domain, which ensures that the receptors remain inactive, until they are bound by an activating RNA. An additional pair of caspase activation and recruitment domains (CARDs) are present at the N-terminus of both RIG-I and MDA5, which mediates downstream signal transduction by interacting with the CARD domain of the mitochondrial membrane-associated protein and MAVS. On the contrary, LGP2 lacks the N-terminal CARD domain and thus is not capable of interacting with MAVS. As a result, LGP2 is believed to regulate the RIG-I and MDA5 signaling pathways rather than acting independently as a signaling receptor. It is been almost 16 years of the discovery that showed RIG-I and MDA5 induces type I interferons (T1-IFN) signaling through overexpression studies. (Yoneyama et al., 2004) Later on, RIG-I and MDA5 knockout studies in a mouse model of virus infection demonstrated that the receptors are essential for T1-IFN production and antiviral defense mechanism (Rehwinkel and Gack, 2020), (Kato et al., 2006)

### RIG-1 Activation

In 2004, Takashi Fujita’s group discovered that RIG-1, a cytoplasmic RNA helicase is responsible for promoting T1-IFN induction upon viral infections (Yoneyama et al., 2004). Later on many research findings showed that RIG-1 is a key PRR in sensing variety of RNA viruses including flaviviruses, alphaviruses, coronaviruses, reoviruses, paramyxoviruses, Orthohantavirus orthomyxoviruses, rhabdoviruses, arenaviruses, and bunyaviruses (Kell and Gale, 2015). RIG-1 has been shown to recognize multitude of PAMPs, including short double-stranded RNA (dsRNA) “containing either a 5′ triphosphate, 5“ diphosphate or 5” monophosphate moiety (Hornung et al., 2006; Goubau et al., 2014; Spengler et al., 2015). It is now increasingly been shown that RIG-1 can also distinguish between the self-RNA from the viral RNA, based on post transcriptional modifications at 5′ triphosphate end of the RNA (Schuberth-Wagner et al., 2015; Devarkar et al., 2016).

In most cell types, RIG-I is expressed minimally. However, its abundance increases when exposed to IFN. RIG-1 contains a number of domains that regulates the sensing of PAMPs and consequently their activation (Kell and Gale, 2015). The Rig-1 protein consists of two N-terminal CARDs, followed by two tandem helicase domains (Hel1, Hel2) separated by an insertion domain (Hel2i). This is followed by a CTD, which is also referred as repressor domain (RD). In non-infected cells RIG-1 remains in its inactive form where the RD covers the RNA binding and helicase domains. RD also inhibits self-association of RIG-1, inhibiting its interaction with MAVS. In this close fit conformation, the CARDs are folded on top of each other in such a way that it keeps the protein in an auto-inhibited state (Saito et al., 2007; Kowalinski et al., 2011). The moment RIG-1 recognizes PAMP RNA, it hydrolyzes ATP and undergoes a conformational change which opens the RNA binding domain for closer interaction with PAMP RNA. The RD interacts with viral RNA and the helicase domains, resulting in the release of the CARDs for MAVS interaction and further signaling. Following the conformational change, both the RD and CARDs undergo posttranslational modifications. The RD is modified through E3 ubiquitin ligase RIPLET, which promotes ubiquitination at N-terminal sites by the TRIM25 protein (Gack et al., 2007; Oshiumi et al., 2010). This allows the CARD–CARD tetramer formation in the presence of ubiquitin. This tetramer of RIG-1 CARDs facilitates the interaction with MAVS and triggers MAVS mediated downstream signaling pathway.

### MDA5 Activation

MDA5 was the first RLR gene that was fully cloned and characterized back in 2002, where the helicase core and CARD domains were found to be responsive to dsRNA (Kang et al., 2002). MDA5 also shows viral sensing for flaviviruses, alphaviruses, coronaviruses, reoviruses, and paramyxoviruses. But the detection of picornaviruses and caliciviruses is predominantly mediated through MDA5 (Kato et al., 2006) However, activation of MDA5 requires higher-order RNA web structures generated during viral infections rather than simply long molecules of dsRNA (Pichlmair et al., 2009). MDA5 thus is also a double-stranded RNA-dependent ATPase, consisting of both CARDs and an almost identical set of RNA helicase and RNA binding motifs. In addition, the CTD has a different function in MDA5 compared to RIG-1. Unlike RIG-1, the CTD of MDA5 has no RNA affinity and instead it is required for cooperative filament assembly (Peisley et al., 2011). However, similar to RIG-1, RNA binding with MDA5 results in the CARDs to interact with MAVS, leading to the transcription of the genes encoding IFNs (Wu et al., 2013). MDA5 senses longer dsRNA species and secondary structures which are usually viral replication intermediates and shows no requirement for terminal di-or triphosphates. For MDA5 activation and filament formation the ATP hydrolysis activity of the helicase domains is required (Motz et al., 2013). Further, these filaments expose the CARDs for interaction with the CARD motif of MAVS. Post translational modifications like protein phosphatase-1 (PP1) dephosphorylation of MDA5 CARDs have also been shown to regulate MDA5 activation (Wies et al., 2013). However, unlike RIG-I, not much is known about other post translational modifications such as ubiquitination that may regulate MDA5 activation. Overall, the underlaying mechanism and the biology about how the regulation and activation of MDA5 takes place has not been investigated at great depths.

### LGP2 Activation

Similar to RIG-1 and MDA-5, LGP2 have also been identified as DExD/H box RNA helicases that function in the antiviral immune response (Rothenfusser et al., 2005). Although LGP2 is less well characterized than the other two. LGP2 lacks the CARD domain and acts as a negative regulator of RIG-1 and MDA5 mediated signaling while sometimes also acts as an enhancer of MDA5-directed signaling (Bruns et al., 2014) (Yoneyama et al., 2005) (Venkataraman et al., 2007). Structurally LGP2 consists of the similar helicase and RD domains like RIG-1 and MDA5 but lacks the N-terminal CARD domains, required for interaction with MAVS. Thus, its effect on downstream antiviral signaling is often due to interaction with dsRNA viral ligand or the other RLRs (RIG-I and MDA5). Uninfected cells express low levels of LGP2 but it accumulates as a result of viral infections (Komuro and Horvath, 2006). LGP2 can recognize various RNAs, irrespective of length or 5′ phosphate ends (Pippig et al., 2009). Although it seems that LGP2 serve multiple, diverse functions in response to different viruses, but there have been conflicting reports regarding LGP2’s function in the immune response depending on the experimental approach taken (Zhu et al., 2014). Therefore, further research is needed to understand the role and function of LGP2 in the regulation of RNA virus sensing and downstream signaling events.

### DNA Sensors in the Cytosol

In 2006, two parallel reports demonstrated that mammalian cells produce IFNs upon detection of cytosolic double-stranded DNA (dsDNA). This spurred the idea that cytosolic DNA sensing is a major mechanism by which the innate immune system detects pathogens (Ishii et al., 2006) (Stetson and Medzhitov, 2006). Afterwards several DNA sensors were identified, but only few have been proven to have a clear and definitive mechanism to induce IFN response to cytosolic DNA release. However, the presence of DNA in the cytosol is not only an indicator of pathogen infection, but it can also render to cellular damage and the cell’s nuclear integrity. As far as pathogenic infections are concerned, with regards to DNA sensing, the cellular defense mechanism is triggered not only for DNA viruses, but also for bacterial and eukaryotic pathogens. While sensors and pathways related to detection of RNA viruses are well defined, most of the sensors of viral DNA have only been recently identified. Furthermore, the signaling pathways that lead to IFN production in response to viral DNA PAMPs are less well defined than pathways activated by viral RNA PAMPs. However, many discoveries in the past decade, have revealed key factors in the DNA-sensing through IFN-stimulatory DNA (ISD) pathway (Stetson and Medzhitov, 2006) (Yanai et al., 2018). The most well studied and relevant receptors in triggering the IFN pathways are the cGAS/STING, DAI and ALR pathway, which will be described in the next sections (Briard et al., 2020).

### cGAS/Sting Pathway Activation

Amongst all the cytosolic DNA sensors, cGAS signaling is probably the most explored pathway. The pathway is triggered during infection with cytosolic bacterial pathogens and some DNA viruses resulting into transcriptional induction of T1-IFNs and the nuclear factor-κB (NF-κB) dependent expression of proinflammatory cytokines. The most common DNA viruses that cGAS senses are herpesviruses, human papillomavirus, adenovirus, and hepatitis B virus, as well as retroviruses such as human immunodeficiency virus-1 (HIV-1), simian immunodeficiency virus and murine leukemia virus (Ma and Damania, 2016). Although cGAS is known to play a role in the innate immune response to a number of positive-sense RNA viruses, the mechanism of RNA sensing and signaling remains largely unexplored (Schoggins et al., 2014).

Structurally cGAS is composed of N-terminal unstructured region and is also less conserved across species, followed by nucleotidyl transferase domain and a C terminal Mab21 domain (Sun et al., 2013). The DNA-sensing mechanism in this system mainly comprise of cGAS which is basically a DNA-sensing nucleotidyl transferase enzyme, its second-messenger product that is cyclic GMP–AMP (cGAMP) and the cGAMP sensor STING (also known as MITA13, ERIS14 or MPYS15,16). At resting state cGAS exists in a bilobal conformation, with a zinc thumb positioned between the lobes. The activation happens by direct DNA binding, which triggers conformational changes to induce the enzymatic activity (Civril et al., 2013) (Kranzusch et al., 2013; Li et al., 2013). Normally any DNA, foreign or self, can cause cGAS activation, but the length of the DNA is crucial for the trigger. Shorter DNA of approx. 20 bp can bind to cGAS, but longer dsDNAs of more than 45 bp can form more stable ladder-like networks of cGAS dimers, which consequently leads to stronger enzymatic activity (Li et al., 2013) (Zhang J.-Z et al., 2014) After the DNA binding, the catalytic pocket of cGAS is accessible for synthesis of cGAMP by converting GTP and ATP into cGAMP. This synthesis generates 2′3′-cGAMP, which is an endogenous cGAMP, containing two unique phosphodiester bonds (Sun et al., 2013; Schoggins et al., 2014) (Zhang X et al., 2013). 2′3′-cGAMP binds STING, which is an endoplasmic reticulum (ER)-localized adaptor. STING can also directly bind to cyclic dinucleotides produced by bacteria, including cyclic diGMP, cyclic diAMP and bacterial cGAMP, all of which have conventional (3′5′) phosphodiester linkages.

Although cGAS can directly bind to the DNA moieties, the production of cGAMP is essential for STING activation to induce T1-IFN. STING activation by cGAMP creates conformational change leading to STING dimerization and is then it is subjected to K63-linked ubiquitination by TRIM56, TRIM32 and MUL1 (Oshiumi et al., 2010; Tsuchida et al., 2010; Zhang et al., 2012; Ni et al., 2017) Further regulation of cGAS activity is governed by post translational modifications. Most post translational modification sites are found on the nucleotidyl transferase domain and on C-terminal domain of cGAS. The most predominant post-translational modifications of cGAS are phosphorylation, ubiquitination, acetylation, glutamylation, and sumoylation that are reported to profoundly affect its function (Wu and Li, 2020).

### DAI Activation

DNA-dependent activator of IFN-regulatory factors (DAI) (also known as ZBP1 or DLM-1) is a cytosolic sensor molecule for dsDNA and is implicated in antiviral responses to some DNA viruses. It was the first cytosolic DNA sensor of antiviral innate immunity to be discovered by Taniguchi group in 2007 (Takaoka et al., 2007). Similar to cGAS pathway the dsDNA-stimulated DAI also activates IRF3 and NF-kB leading to the production of type-I interferons and inflammatory cytokines (Takaoka et al., 2007). Predominantly DAI can sense viruses including herpes simplex virus-1 (HSV-1), human cytomegalovirus (HCMV), mouse cytomegalovirus (MCMV) and Human immunodeficiency virus (HIV) (DeFilippis et al., 2010; Hayashi et al., 2010; Upton et al., 2019). Apart from recognizing DNA viruses, DAI is able to sense self-DNAs in the cytosol, which plays a crucial role in the development of autoimmune diseases like Systemic lupus erythematosus (SLE) (Zhang W et al., 2013). Increased DAI expression has been shown in SLE patients, where activation of DAI mediated by calcium signaling results in pathological macrophage activation in SLE disease (Zhang W et al., 2013). Another recent study shows that DAI also is involved in caspase independent cell death called necroptosis. This is induced by E3-Zα-domain-deleted vaccinia virus (VACVE3LΔ83N) (Koehler et al., 2017). Some reports also suggests that DAI plays a critical role in the activation of the NLRP3 inflammasome in Influenza A virus (IAV) infected bone marrow-derived macrophages (Kuriakose et al., 2016). In the same line of investigation a recent study shows that DAI is capable of sensing Z-form RNAs produced during IAV infection, culminating into necroptosis (Zhang et al., 2020).

The overall mechanism by which DAI senses cytosolic DNA is scarcely known. Some studies have shown that DAI binds to DNA in a length-dependent manner, but is independent of sequence specificity. The DNA serves as a scaffold to mediate the formation of a tandem array of DAI molecules, which then recruit and activate downstream signaling molecules, such as TBK1 and IRF3 (Wang et al., 2008). Extensive research is needed to gain a better understanding of the underlaying mechanisms through which DAI senses DNA. There are no clear reports which demonstrates whether or not DAI signals through STING pathway (Radoshevich and Dussurget, 2016)-(Xu et al., 2015)Future studies to understand its role, mechanistic action and contribution in manifestation of innate immune response in variable infections needs to be thoroughly investigated.

### AIM2-like Receptors (ALRs) Activation

The ALRs also participate in the detection of intracellular DNA and acts as sensors of the ISD pathway. They are known to activate inflammasomes in response to infections due to pathogens (Hornung et al., 2009). Since 2009 at least 10 or more proteins have been proposed as cytosolic DNA sensors. To name a few AIM2, IFI16, LRRFIP1, DHX9, DHX36, DDX41, Ku70, DNA-PK, MRE11, cGAS, STING and Rad50. However, it is only AIM2 and IFI16 that have been shown to detect viral dsDNA in the cytoplasm by direct binding via the AIM2 HIN200 domain. This further mediates inflammasome and transcription factor activation (Dempsey and Bowie, 2015) (Johnson et al., 2013) (Rathinam et al., 2010). During inflammasome activation, ASC (apoptosis-associated speck-like protein containing a CARD) brings caspase-1 to the inflammasome complex by CARD-CARD interactions. Activated caspase-1 then leads to the induction of a cell death pathway that is stimulated by a range of microbial infections called pyroptosis. This is mediated via the proteolytic cleavage of the N-terminal domain of gasdermin D that generates pores on the host cell membrane from which the proteolytically cleaved form of proinflammatory cytokines IL-1β and IL-18 are released (Sharma et al., 2019). Other than activating inflammasomes, IFI16 is also involved in activating the ISD pathway by sensing non-self DNA in both the nucleus and cytosol (Kerur et al., 2011). IFI16 has also been reported in regulating cellular transcription and act as a DNA virus restriction factor. IFI16 knockdown disrupts the latency of Kaposi’s sarcoma associated herpesvirus (KSHV) and induced lytic transcripts (Roy et al., 2016). Mechanistically, IFI16’s is reported to have an interaction with H3K9MTases leading to epigenetic silencing of foreign DNA (Roy et al., 2019). While these studies implicate IFI16 as an important sensor of both cytosolic and nuclear foreign DNA, several other reports suggest contradictorily. For example, in one study it was shown that ALRs are not required for the T1-IFN response to transfected DNA, DNA virus infection, or lentivirus infection. Moreover, IFI16 in primary human fibroblasts was shown to be dispensable for the ISD response to transfected DNA and HCMV infection (Gray et al., 2016). However, cGAS knockout cells did not generate an effective T1-IFN response. On the other hand, this study demonstrated the importance of cGAS as the primary DNA sensor in the ISD pathway. In conclusion, DNA sensing pathways like DAI ALRs like IFI16 may have a very specific cell type specific role or probably a redundant function in triggering ISD pathway. Therefore, the uncertainty that still thrives in this field of DNA sensors, needs to be studied at large to have deeper and clear understanding.

### The Release of mtDNA

Although a lot is known about the role of mitochondrial nucleic acids in eliciting a pro-inflammatory response, the detailed mechanism of the actual release of the nucleic acids is highly elusive. A wide range of pathologies and infections are known to initiate the release of predominantly oxidized mtDNA into the cytoplasm triggering the recognition by sensors such as cGAS (West et al., 2015). TLR4 activation in experimental Autoimmune Myocarditis leads to significant amounts of circulating mtDNA in mice (Wu et al., 2017). They demonstrate a clear need for the circulating DNA to be oxidized. They also show that TLR4 activation induces ROS stress which may promote cardiomyocytes mtDNA damage and increase circulating mtDNA levels. Melatonin, which is synthesized by neuronal mitochondria and acts as an endogenous free radical scavenger, decreases with age and neurodegeneration. Studies with melatonin-deficient aralkylamine N-acetyltransferase (AANAT) knockout mice place ROS damage induced mitochondrial dysfunction as an initiating event for mtDNA release (Jauhari et al., 2020). Furthermore, infections with RNA viruses such as the dengue virus or bacterial pathogens such as *Mycobacterium tuberculosis* have been shown to increase mitochondrial stress and causes the release of mtDNA (Wiens and Ernst, 2016; Sun et al., 2017). It has been shown that various pathogens can induce limited mitochondrial membrane permeabilization called minority MOMP (Brokatzky et al., 2019). Under sub lethal stress conditions, minority MOMP triggers genomic DNA instability and engagement of mitochondrial apoptotic signaling.

Another interesting mechanism of mitochondrial nucleic acid release is found in breast cancer cells (Rabas et al., 2021). PINK1 association to mitochondria in metastatic cancer cells, promotes mitophagy and generation of extracellular vesicles in these “donor” cells. Thus, invasive characteristics are transferred to the “recipient” tumor cells. The key cargo in the vesicles is the mtDNA which activates TLR9 “recipient” tumor cells leading to increased endosomal trafficking which finally potentiates carcinoma progression. On the other hand, cancer cells also use similar mtDNA laden extracellular vesicles to target and induce the production of IFN and IL-6 from macrophages, which attenuates T-cell immunity in the tumor micro environment, thus promoting tumorigenesis (Cheng et al., 2020). They found oxidized mtDNA to be released into the cytosol when Lon is overexpressed. However, the mechanism of the DNA release and packaging into extracellular vehicles is still not clear.

A clear mechanism of mtDNA release is observed during programmed cell death. Studies have shown that under conditions of BAX and BAK mediated outer membrane permeabilization, the caspase inhibition can cause the pores to increase considerably in size (McArthur et al., 2018; Riley et al., 2018). This allows inner membrane herniation and extrusion of mtDNA and dsRNA. It is still not clear if the herniated inner membrane forms a vesicle around the mitochondrial nucleic acids and continues to exist as vesicles in the cytoplasm. The other possibility is that such herniated structures are unstable and lead to release of the mitochondrial nucleic acids directly in the cytoplasm. Additionally, studies have also shown depletion of mitochondrial helicase SUV3 and polynucleotide phosphorylase (PNPase) leading to dsRNA release from mitochondria through BAX/BAK mediated outer membrane permeabilization (Dhir et al., 2018). One recent study further supported the mitochondrial herniation model. Here, mitochondrial TALENs were used to induce mtDNA breaks. This resulted in BAX/BAK mediated mtRNA release and activation of RIG-1/MAVS sensors (Tigano et al., 2021). Thus, it is very clear that apoptotic caspase activation must be shut down for hijacking the pore forming machinery to initiate mitochondrial nucleic acid mediated inflammation.

Another plausible mechanism is the engagement of the membrane permeability transition pore mPTP (Ricchelli et al., 2011). In many non-apoptotic models, the mPTP has been shown to be instrumental in release of mtDNA fragments (Kim et al., 2019). The study used cells lacking the mitochondrial endonuclease G to show release of 100–200 bp fragments. Another recent study has established the release through mPTP in YME1L deficient cells (Sprenger et al., 2021). All the studies which rely on this model show that VDAC oligomerization inhibitor, VBIT4 specifically blocks any mitochondrial nucleic acid release in these conditions (Yu et al., 2020; Torres-Odio et al., 2021). It is still not clear how a pore predicted to pass molecules smaller than 1.5 kDa is able to transport the entire mtDNA nucleoid. It could be possible that chronic opening of the pore causes swelling of the mitochondria and thus leads to a bigger pore size allowing efflux of nucleic acids.

Thus, in conclusion, more studies in different cell types and model organisms are necessary to establish the model and mechanics of mitochondrial nucleic acid release.

### Signaling Pathways Triggered by Mitochondrial Nucleic Acid Sensing

mtDNA and mtRNA are dependent on different receptors and adaptors, but there is considerable overlap between the downstream signaling afterwards. This is evident from the fact that the final IFN response was attenuated only in double deletion of MAVS and STING both and not in situations where the individual gene were deleted (Brunette et al., 2012). Thus, sensing either of the nucleic acids leads to similar gene expression outcomes.

Following recognition of the mtRNAs, the PRRs activate the downstream signaling of the antiviral innate immunity pathway. The key player immediately downstream of PRRs is the mitochondrial antiviral signaling protein (MAVS). Upon activation, MAVS undergoes aggregation to form multimeric filaments (Hou et al., 2011). This filamentous form of MAVS is a platform on which other proteins can dock. At this point the signaling pathways splits into two molecular cascades. The first cascade relies on proteins Tank binding kinase-1 (TBK1) and IκB kinase epsilon (IKK ɛ). Both being serine/threonine kinases, phosphorylates the transcription factors IRF3 and IRF7 to trigger their dimerization and nuclear translocation (Hiscott, 2007). Once inside the nucleus, IRF3 and IRF7—which have great structural homology to each other—mediate the expression of type I and type III IFNs (Jefferies, 2019). The canonical interferon response element sequence (IRES) in the promoter of IFN-β and IFN-α is the binding site for IRF3 after its association with the co-activator CREB-binding protein (Jing et al., 2020). Similarly, route of homodimerization is adopted by IRF7 for its function as a transcription factor (Honda et al., 2005). Going back to the second cascade of MAVS signaling, the IKKα/β/γ complex is employed for NF-κB dependent upregulation of proinflammatory genes (Fang et al., 2017). Autocrine and paracrine responses, through the IFN-a/b receptor, is the ultimate result of T1-IFN production. This leads to the transcription of hundreds of IFN-stimulated genes (ISGs) through the activated JAK/STAT signaling pathway (Nan et al., 2017).

Recognition of the mtDNAs is signaled through cGAS-STING pathway. As discussed in the aforementioned section of cGAS activation, STING upon activation is then able to bind TBK1. Together they translocate to perinuclear endosomes via the Golgi (Zevini et al., 2017). Here in association with TBK1, STING interacts with and activates IRF3, thus leading to T1-IFN (Tanaka and Chen, 2012). STING is also able to cause NF-κB phosphorylation, nuclear translocation, and target gene expression (Abe and Barber, 2014). Thus, eliciting a similar response to that of mtRNA recognition.

## Patho-Physiology of Nucleic Acid Sensing

### mtDNA in (Auto) Immune-Diseases

The aforementioned signaling pathways represent a link between mtDAMPs and innate immune response leading to pathophysiology of autoimmune diseases. The common effect triggered by the PRRs is the release of a type 1 interferons (Hu et al., 2019), which has been reported over the years with increasing frequency in the context of various autoimmune diseases. Therefore, it is necessary to discuss the influence of mtDNA in the pathophysiology of Autoimmune diseases.

Most of the described phenotypes in such cases are related to one of the following events: 1. an excess release of mtDNA due to mitochondrial damages, 2. defective cytosolic nucleic acid degradation methods, 3. defective elimination of damaged mitochondria, 4. mutations in regulatory or stimulating molecules that contribute directly in the induction of interferons, especially by leukocytes. Thus, in the next sections we examine each disease individually and try to pin point which of the above mentioned events contribute to the pathology respectively.

### Role of mtDNA Sensing in Systemic Lupus Erythematosus (SLE)

Kim et al. were first to show a direct connection between the recognition of cytosolic mtDNA, released by the engagement of the membrane permeability transition pore, and Lupus-like disease phenotype manifested by the production of Antinuclear Antibodies (Kim et al., 2019). The cGAS/STING pathway highlighted in this study appears to be associated with SLE and lupus-like interferon-associated diseases.

Another illustrative example of the pathogenesis of SLE by dysregulated DNA sensing mechanisms is that of Aicardi-Goutières syndrome (AGS). AGS is a hereditary systemic inflammatory disease which is characterized by overexpression of IFN1 (Crow and Manel, 2015). Here mutations, that are also often found in SLE, particularly affect various DNA sensing molecules. The excessive degradation of nucleic acids can lead to the expression of a lupus phenotype (Crow et al., 2006; Lee-Kirsch et al., 2007; Namjou et al., 2011). The phenotype seems to be closely related to the induction of interferons through the cGAS-STING pathway, as the deletion of involved factors led to a recovery of the phenotype (Stetson et al., 2008; Yan et al., 2010; Gall et al., 2012; Ablasser et al., 2014; Ahn et al., 2014). Similarly, an increase in STING activity also led to lupus-like symptoms and interferon induction (Jeremiah et al., 2014; Liu et al., 2014).

### Proinflammatory Potential of Extracellular mtDNA in NETosis

Cell-free mtDNA released in the plasma plays a critical role in a recently addressed aspect of SLE—the formation of Neutrophil Extracellular Traps. This process is the ability of Neutrophils to release nucleic acids—also mtDNA—together with antimicrobial enzymes, as a first line defense mechanism against bacterial infection (Brinkmann et al., 2004; Wang et al., 2015). Physiologically, the factor TFAM gets activated by PKA and associates with pro-inflammatory oxidized mtDNA. This triggers its lysosomal degradation. However, dysfunction in the degradation of oxidized mtDNA, causes induction of type 1 interferons in leukocytes (Caielli et al., 2016). In SLE patients, PKA was found to be less active. This leads to NETs containing higher amounts of oxidized mtDNA (Lood et al., 2016). However, release and reaction to mtDNA is not only limited to Neutrophils. It was shown, that also Eosinophils and lymphocytes might trigger type 1 interferon response by releasing mtDNA in to the plasma (Yousefi et al., 2008; Ingelsson et al., 2018). The activation of leukocytes seems to be mediated mainly by TLRs. Interestingly, the NET formation seems to be also stimulated by cell-free mtDNA via TLR9 (Zhang et al., 2010), observed during primary graft dysfunction after lung transplantation. The authors hypothesized, that ischemic conditions might trigger the release of mtDNA which in turn led to the activation of neutrophils, causing increased NET formation resulting in lung injury (Mallavia et al., 2019).

All in all, the elevated concentrations of mtDNA in Plasma SLE patients, led to mtDNA being used as a possible new biomarker for SLE. This new biomarker not only correlates with the severity of the disease, but also with the development of a Lupus Nephritis (Truszewska et al., 2020).

### MtDNA in Other Autoimmune Diseases

In addition to SLE, other autoimmune disorders are also highly related to an overactive IFN 1 response often in correlation with release and sensing of mtDNA. A group of diseases were termed type 1 interferonopathies, due to their origin of dysregulation of the type 1 interferon pathway. These include ISG15-and DNAse2 deficiency or AGS (described above), just to name a few. In conclusion this emphasizes the importance of mtDNA sensing as a possible trigger for an interferon induction in Autoimmune diseases.

### Rheumatoid Arthritis

The pro-inflammatory potential of oxidized mtDNA in Rheumatologic diseases can be seen from the study where intra-articulary injection of oxidized mtDNA in mice caused progression of arthritis by stimulation of macrophages and induction of NF-ΚB (Collins et al., 2004) Moreover, circulating mtDNA was found in plasma and synovial fluid of RA patients ((Hajizadeh et al., 2003). Additionally, when there is dysfunction of DNAse I in apoptotic cells, there is insufficient degradation of extranuclear DNA. This leads to T1-IFN inflammation and arthritis in mice (Rodero et al., 2017). Thus, elimination of such apoptotic cells by macrophages might trigger a dysregulated systemic immune response through stimulation of cGAS, AIM2 and TLRs (Ahn et al., 2012; Baum et al., 2014; Jakobs et al., 2015).

Li N et al. (2019) demonstrated an important mechanism in the contribution of mtDNA in pro-inflammatory mediated CD4^+^ T cells RA (Li Y. et al., 2019). It was shown, that defect of the mtDNA repair nuclease MRE11A, which seems to be associated in the progression of RA in humans (Li et al., 2016), caused not only leakage of mtDNA into the cytosol, but also its recognition by AIM3 and NLRP3. This leads to the stimulation of inflammasome, caspase1 and pyroptotic cell death. The phenotype was confirmed *in vivo*, showing aggressive tissue inflammation, caspase one activation and mtDNA accumulation in synovial tissue (Li Y. et al., 2019). Strikingly, not only does the interferon response appear to be conditioned by mitochondrial dysfunction, but T1-IFN itself also seems to worsen mitochondrial function. This self-reinforcing feedback occurs via suppression of NRF2 by interferon signaling, resulting in increased oxidative stress and enhanced proinflammatory cytokine responses (Lei et al., 2021).

Because increased levels of cell-free mtDNA are also associated with several other inflammatory diseases—e.g., granulomatosis with polyangiitis (Hashimoto et al., 2021), further intensive research on the pro-inflammatory role of mtDNA should be conducted.

### mtDNA in Other Chronic Diseases

Apart from systemic inflammatory diseases, mitochondrial nucleic acid sensing seem to play a central role in the pathophysiology of the many organ specific inflammatory conditions. In the following sections we will focus on these pathologies. We would also like to distinguish them from the effects of circulating mtDNA, which may result from acute tissue damages in these organs.

### mtDNA in Neuroinflammatory Diseases

#### Parkinson’s Disease

The influence of mitochondrial DNA on neuroinflammatory diseases has been most clearly demonstrated in Parkinson’s disease (PD), a disease in which motor activity dysfunction results from the degeneration of dopaminergic neurons in the substantia nigra. Characteristically for pathology of PD, the so-called Lewy (protein) bodies are deposited in the affected brain areas and secretion of various cytokines indicate an inflammatory component of the disease (Mogi et al., 1994; Dobbs et al., 1999).

Early on, a link was recognized between defective mitophagy, the mechanism to eliminate malfunctioning mitochondria, and the pathogenesis of Parkinson’s disease and neuroinflammation. Mutations in Parkin/PINK1, two key players in mitophagy, coordinating the lysosomal degradation by ubiquitination of the mitochondrial outer membrane proteins, were associated with a PD phenotype in several cell models (Abbas et al., 1999; Lücking et al., 2000; Valente et al., 2004; Lazarou et al., 2015; Wauer et al., 2015; Gladkova et al., 2018). However, the correlation between dysfunction of autophagy and development of PD was shown mainly *in vitro* and not *in vivo* experiments (Goldberg et al., 2003; Perez and Palmiter, 2005; Kitada et al., 2009). Thus, it looks like just the mutations are not sufficient to elicit the complete pathology of the disease. Recent studies shed new light on this aspect. In addition to the mutation in Parkin/PINK1, a second proinflammatory stimulus seems to be required to induce the onset of the disease *in vivo*. Sliter et al. showed that Parkin/PINK1 deficient mice at rest had no pathological differences in characteristic inflammatory cytokine levels (IL6, IFN b and circulating mtDNA). In contrast, by either exercise of the Parkin/PINK1 deficient mice or mice gathering further mtDNA mutations, the inflammatory stimulus was measured. Interestingly, this inflammatory response seemed to be strongly dependent on STING pathway and thus interferons (Sliter et al., 2018). This was based on the observations that, deletion of STING or receptor blockade of Interferon-alpha/beta receptor (IFNAR), respectively, completely prevented the release of cytokines. Recently, new evidence for the involvement of mitochondrial DNA in this pathophysiology has been revealed. The absence of PINK1, but also other autophagy molecules GBA and ATP13A2, led to accumulation of mtDNA in the cytosol and consequently to IFN1 induction in cultured neuroblastoma cells. This phenotype was completely prevented by overexpression of DNAse II, but also by depletion of IFI16, an mtDNA sensor molecule. Strikingly, DNAse II overexpression also improved symptoms in the zebrafish animal model of PD. A subsequent post-mortem analysis of PD patients showed an increase in cytosolic mtDNA and IFI16 levels in the medulla oblongata. It was striking that IFI16 was particularly associated with the Lewy bodies specific for the disease (Matsui et al., 2021).

Similar to other diseases, in PD circulating mtDNA was described, too. It seems to decrease due to affective treatment and thus can be discussed as a possible biomarker (Lowes et al., 2020).

#### Amyotrophic Lateral Sclerosis

Amyotrophic lateral sclerosis (ALS) is a neurodegenerative disease effecting the motor neurons. The disease pathology which is also connected to TLR signaling, interleukin release and activation of the inflammasome. A pathological marker for ALS is the accumulation of TDP-43 in the cytosol. This had been associated with interferon and NF-ΚB inflammatory signaling. Recently, it was shown, that mitochondrial DNA was released through mPTPs into the cytosol after TDP-43 treatment (Yu et al., 2020). This resulted in induction of interferons through NF-ΚB signaling. However, this could be prevented by deletion of cGAS/STING in cells and mice models. Furthermore, higher amounts of signaling intermediates of this pathway found in ALS patients spinal cord samples indicated, that mtDNA derived signaling might play a significant role in pathogenesis of ALS. Involvement of the cGAS-STING pathway and its activation by released mtDNA in neuroinflammation seems likely, which could be triggered by the absence of the antioxidant melatonin in a mouse line and was found in association with Huntington’s disease (Jauhari et al., 2021).

#### Visual System

Mitochondrial dysfunction is often connected to pathologies associated with eye-related inherited disorders (Yu-Wai-Man and Newman, 2017). Recently, evidence for mtDNA induced inflammation in the visual system was shown in the experiments where cultivated retinal microvascular endothelial cells released mtDNA in response to oxidative stress (Guo et al., 2020). Also, in rat models, intravitreal injection of LPS and light injury in the retina triggered the release of mtDNA in retinal tissue (Guo et al., 2021). In both studies, the released mtDNA triggered the cGAS/STING pathway resulting in an T1-IFN upregulation. This, supports the need to investigate the significance of mitochondrial nucleic acid sensing in retinal neurodegeneration.

The central importance of mitochondrial DNA-triggered inflammation was also shown in studies of neuromyelitis optica - an autoimmune disease characterized by aquaporin four autoantibody-mediated damage to astrocytes. As a consequence, there are not only visual disturbances but also sensorimotor deficits. Shimizu et al. showed that astrocytes, upon treatment by the autoantibodies, released proinflammatory cytokines (CCL2) and mtDNA. The activation of TLR9, on the one hand increased the recruitment of monocytes, on the other hand mtDNA through *feedback loop* stimulated the further production of CCL2 (Shimizu et al., 2020). This indicates a central role of mtDNA in the pathophysiology in neuromyelitis optica.

Finally, mitochondrial autophagy regulators like Parkin/PINK1 seem to be crucial for retinal degeneration diseases, as they were reported to protect retinal photoreceptors from oxidative stress (Zhou B et al., 2021).

### Role of mtDNA in Liver Disorders

The two important examples of a diseases strongly related to mitochondrial dysfunction are non-alcoholic fatty liver disease (NAFLD) and non-alcoholic steatohepatitis (NASH). Both these conditions are precursor of hepatocellular carcinoma (HCC). HCC represents a central problem in industrialized countries with increasing numbers of cases. Although mitochondria also appear to be involved in dysregulated lipid metabolism in NAFLD (Longo et al., 2021), here we focus predominantly on pathologies triggered by mitochondrial nucleic acids.

The difference between NAFLD and NASH is mainly the inflammatory and fibrotic component of the later disease. Kupffer cells and macrophages are the main causative agent of fibrosis (Hirsova and Gores, 2015; Hirsova et al., 2017; Yuan et al., 2017). cGAS-STING signaling in Kupffer cells and macrophages might contribute in the development of NASH and fibrosis. It was found to be activated in hepatic tissue samples from NAFLD patients as well (Luo et al., 2018). Nonspecific induction of STING in macrophages and STING-IRF3 signaling, caused hepatic inflammation, steatosis and fibrosis. On the other hand, STING depletion ameliorated these consequences (Iracheta-Vellve et al., 2016; Luo et al., 2018; Qiao et al., 2018).

Strikingly, Yu et al. showed that mtDNA released by hepatocytes caused the secretion of TNF-a and IL-6 by Kupffer cells, which was attenuated by STING deletion. In addition, deletion of STING prevented the progression of hepatic steatosis (Yu et al., 2018). Furthermore, mtDNA can also directly activate hepatic stellate cells and push them to the progression of fibrosis (An et al., 2020). Also, in pathophysiology of NAFLD, the potential induction of TLR9 signaling by mtDNA seems to play a role, since Garcia-Martinez et al. were the first to show a direct link between the release of oxidized mitochondrial DNA from hepatocytes and its recognition by TLR9 in humans and in mouse model (Garcia-Martinez et al., 2016). This is supported by the observation that TLR9 deficient mice did not develop NASH on a provocative diet (Miura et al., 2010).

Thus, it becomes tempting to speculate, that the damaged hepatocytes are the drivers of mtDNA release, triggering the Kupffer cells and hepatic macrophages to induce pro-inflammatory and pro-fibrotic pathways.

### Role of mtDNA in Pulmonary Diseases

The cell-free mtDNA concentrations changes with different pathologies associated with lungs. In case of acute lung injury the amounts go up, correlating to the extent of damage, whereas in lung carcinoma the amounts go down (Chen et al., 2018; Mao et al., 2021). This already indicates a possible involvement of mtDNA release and sensing in different pathologies of the pulmonary system.

Several independent studies indicate different paths leading to the same root cause. An analysis of samples from lung fibrosis patients showed higher levels of mtDNA mutations and dysfunction of the respiratory chain, indicating an involvement in fibrotic pathways (Jaeger et al., 2019). However, the authors did not address the contribution of mtDNA sensing pathways in these samples. This correlation was investigated in other studies, showing an induction of inflammatory pathways due to cell free mtDNA or cytosolic mtDNA. The triggering the of TLR9/NF-ΚB expression and the activation of the inflammasome pathway was observed in both, cultivated macrophages and lung tissue samples in mice (Zhang X et al., 2014; Wu et al., 2019). Injection of mtDNA caused the secretion of proinflammatory cytokines (IL-1b, IL18, TNFα) as well as the activation of Caspase -1. Both are associated with fibrotic and acute pulmonary injuries (Jäger et al., 2021).

While searching for possible triggers for mtDNA release, it was discovered that H_2_O_2_ released by *Streptococcus pneumoniae* caused severe mitochondrial- and histopathological damage. This, subsequently led to the release of mtDNA into the cytoplasm of human alveola cells. Further, an T1-IFN response was triggered, in which STING was found to be involved (Gao et al., 2019). Furthermore, ZBP1 is proposed to be involved as a mtDNA sensor and mediator of an interferon response (Szczesny et al., 2018). Sustained low level oxidative stress caused damage in the mtDNA of cultured pulmonary epithelia cells, but not the nuclear DNA. As a consequence, mtDNA was released into the cytosol and ZBP1 initiated T1-IFN response *via* TBK1. Interestingly, it was also shown that mtDNA was released extracellularly by exosomes, which also elicited an inflammatory response in healthy neighboring cells, suggesting an autocrine as well as a paracrine potential of mtDNA in lung pathologies.

### Role of mtDNA in Kidney Diseases

Several kidney-related diseases including in diabetes, tubulo-nephritis show elevated mtDNA levels not only in the plasma, but also in the urine. Thus, it is important to discuss the role of the kidney in the involvement of elimination of potentially proinflammatory cell free mtDNA and look closely at the effect of mtDNA on the kidney itself (Whitaker et al., 2015; Wei et al., 2018; Chang et al., 2019).

As in other organ systems, TLR9, as well as cGas-STING signaling triggered by released mtDNA, seem to mediate inflammatory responses in acute kidney injury (Tsuji et al., 2016; Maekawa et al., 2019). STING mediated sensing of mtDNA might also be involved in kidney fibrosis, especially by triggering NF-ΚB, shown in renal cells of TFAM knockout mice. Since suppressing STING pathway ameliorated kidney fibrosis in mouse models of chronic kidney disease, it should be discussed as a possible target in fibrosis treatment strategies (Chung et al., 2019). Furthermore, activation of NLRP3 Inflammasome, which was reported in association with mitochondrial dysfunction was linked to renal tubular injury and tubulointerstitial fibrosis (Gong et al., 2016; Guo et al., 2017).

### Role of mtDNA in Cardiovascular Diseases

The inflammatory potential of mtDNA is connected to different cardiac phenotypes, as extracellular mtDNA, which was found in higher concentrations in association with various cardiac pathologies. It was shown to activate NF-ΚB *via* TLR9 signalling in cardiomyocytes, even inducing its cell death (Bliksøen et al., 2016; Wu et al., 2017). One of the reasons for elevated circulating mtDNA levels can be cardiomyocyte necrosis. For example, in acute myocardial infarction (AMI) (Qin et al., 2017; Nakayama and Otsu, 2018), or ROS-dependent sepsis induced mitochondrial damages (Yao et al., 2015) cardiomyocyte necrosis led to mtDNA release. mtDNA sensing through both, TLR9 and the cGAS-STING pathway must be highlighted as a potential driver of essential pathogenecity in pressure overload-induced heart failure (Hu et al., 2020). In response to pressure overload, the cGAS deficient mice showed not only lower inflammatory cardiac reactions, but more importantly also preserved LV contractile function. In these models, pathological remodeling—including cardiac hypertrophy, fibrosis, and apoptosis—was also very low (Hu et al., 2020).

STING signaling especially in infiltrating macrophages, triggered by mtDNA, might also be involved in inflammation after MI. This provokes IFN stimulation, cardiac expression of inflammatory cytokines and increase of cardiac inflammatory cell infiltration. After MI, the inhibition of IFNAR and IRF3 in mice attenuated ventricular dilation, improved left ventricular dysfunction and survival (King et al., 2017) Supporting its role in pathophysiological involvement in cardiac diseases, Li et al. reported that knockout of STING in mice treated with LPS (mimicking sepsis-induced cardiomyopathy) improved survival rate and cardiac function. Serum and myocardial cytokine levels were decreased and the knockout prevented the apoptosis, as well as NLRP3 mediated pyroptosis of cardiomyocytes (Li N. et al., 2019). Atherosclerosis is one of the major risk factors for MI. It was observed that, mtDNA damage in macrophages and smooth muscle cells (Yu et al., 2013), T1-IFN signaling (Goossens et al., 2010) and inflammasome activation seem to promote atherosclerosis (Duewell et al., 2010). Furthermore, STING-IRF3 pathway triggered endothelial inflammation *via* ICAM-1 in response to the release of mitochondrial DNA provoked by free fatty acids. This not only supported the importance of STING in several inflammatory pathways, but might also display an important connection between cardiovascular pathologies and metabolic syndromes (Mao et al., 2017).

### Rising Knowledge of mtRNA in Pathophysiology

Although the role of mtDNA has been reported more extensively and further findings in this area appear to be of paramount importance, there is increasing evidence for a similar influence of mtRNA. mtRNA was identified as the main potential trigger of the innate T1-IFN immune response (Dhir et al., 2018). Physiologically, cytosolic dsRNA is contained in viruses as well as in mitochondria. But it is also formed to some extent in the cytosol in healthy individuals. It is degraded by a complex of SUV3 and PnPase, which is therefore called the degradosome (Szczesny et al., 2009, 2011; Borowski et al., 2012; Kim et al., 2018; Kotrys and Szczesny, 2019). This process is fundamental not only for defense against viral infections, but also to prevent excessive accumulation of mitochondrial dsRNA in the cytosol. Therefore, dysfunction of the complex also leads to an increased concentration of dsRNA in the cytosol of the affected cell (Pajak et al., 2019). Dhir et al. showed for the first time that, dysfunction of the degradosome and thus increased cytosolic mtRNA can trigger an interferon response via recognition by PRRs (Dhir et al., 2018). The most important receptors for recognition of foreign RNA, as described few sections before, are MDA5, RIG1, TLR3 and PKR (Chattopadhyay and Sen, 2014; Wu et al., 2014; Kim et al., 2018; Linder and Hornung, 2018). However, this mechanism seems to be far more complex, as Brachène et al. pointed out in their work on pancreatic beta cells, investigating the origin of an T1-IFN response observed in pancreatic islet cells in diabetes type 1 (Foulis et al., 1987; Brachène et al., 2021). Apparently, the accumulation of mtRNA in the cytosol is highly dependent on the proliferation state of the cell. Moreover, the induction of an T1-IFN response triggered by cytosolic mtRNA is by no means a general, ubiquitous mechanism of the innate immune system, but is conditioned by cell type (Brachène et al., 2021).

Since it appears that mitochondrial dsRNA (mtdsRNA), like mtDNA, can elicit an interferon response, it is not surprising that increasing evidence also points to a link between (autoimmune) diseases and mtdsRNA—although this is much less understood than for mtDNA. The effects of excessive cytosolic mtRNA can be seen in the study of Zhou et al., who discovered induction of NF-ΚB pathway by MDA5 upon detection of higher cytosolic concentrations of mtRNA (Zhou X et al., 2021). Excessive release of mtRNA was triggered by oxidative stress, provoked by common mtDNA deletions in collagen producing keratocytes. As a consequence, key signalling pathways such as the induction of IL8, key mediator of neutrophil immigration, and profibrotic molecules also appeared to be affected. Although this is a very specific example in keratocytes, it seems tempting to speculate that similar mechanisms could be at work in other organ systems, triggering local proinflammatory and fibrotic remodelling processes.

In addition, dsRNA as such might show proinflammatory effects in Myasthenia gravis (MG). MG is an autoimmune disease which is defined by the development of auto-antibodies against the AChReceptor, thus effecting the motoric nerve system (Cufi et al., 2012). An injection of dsRNA mimicking polyinosinic–polycytidylic acid [poly (I:C)] caused an activation of TLR3, PKR and induction of IFN beta in healthy mice. The analysis of human MG thymus cells further supported the hypothesis of dsRNA’s role in the MG. Most interestingly, the injection of poly (I:C) also provoked a production of auto-antibodies also in wild type mice. This connection was likely strictly related to the interferon pathway, as the antibodies did not develop in T1-IFN Receptor deficient mice. However, this study did only show the pathophysiological potential of dsRNA in general, and not the mitochondrial dsRNA specifically. Apparently, the magnitude of the immune response as well as the organ manifestations depends on the circulating amount of dsRNA (McGarry et al., 2021).

MtdsRNA was found in higher concentration in circulating extracellular vesicles in Alzheimer’s disease. These could be secreted by astrocytes, microglia and neurons under cellular stress (Kim et al., 2020). This might be an indication for considering circulating mtRNA as a potential biomarker for several diseases. However, the role of circulating mtdsRNA remains unclear and it is not investigated, whether it contributes to a systemic inflammatory response or if it is just a consequence of a damaged organ system. A look at the pathophysiology of autoimmune diseases suggests that mtdsRNA may also be involved in the progression of systemic immune responses.

Dermatomyositis is a disease that particularly attacks the striated muscle and skin. Besides intramuscular inflammation, hallmarks in diagnostics of this disease are the induction of interferon inducible genes—such as MHC1, ISG15, and RIG1—n muscle biopsies. There seems to be a strong link between hypoxic conditions and the expression of especially RIG1 in dermatomyositis. Thus, making it a potential inducer of the dysregulated immune response (Luna et al., 2017). Interestingly, mitochondrial transcription seems to be decreased under conditions of hypoxia and thus the amount of mtdsRNA is also reduced (Arnaiz et al., 2021). This represents a direct regulatory mechanism of the immune response triggered by RIG1 induction. Furthermore, dsRNA receptors were found in high expression in skin samples and keratinocytes from psoriasis patients (Rácz et al., 2011). Anti-MDA5 antibodies are found in association with idiopathic inflammatory myopathies, often indicating a lethal prognosis (Li et al., 2014). 40–50% of SLE patients are characterized by dsRNA antibodies (Schur et al., 1971; Davis et al., 1975). Although the development of auto-antibodies is a concept that is not understood in depth and require further investigation, specific auto-antibodies are crucial in diagnostics and prognosis of the diseases.

Finally, it was recently suggested, that mtRNA release might be linked to mtDNA integrity, as mtDNA double strand breaks triggered the release of mtRNA which caused activation of RIG1-MAVS dependent signaling (Tigano et al., 2021). Thus, in conclusion, the field of mtRNA sensing is a very promising for understanding the disease pathology, development of biomarkers and possible therapeutics.

## Discussion and Perspectives

**FIGURE 4:**
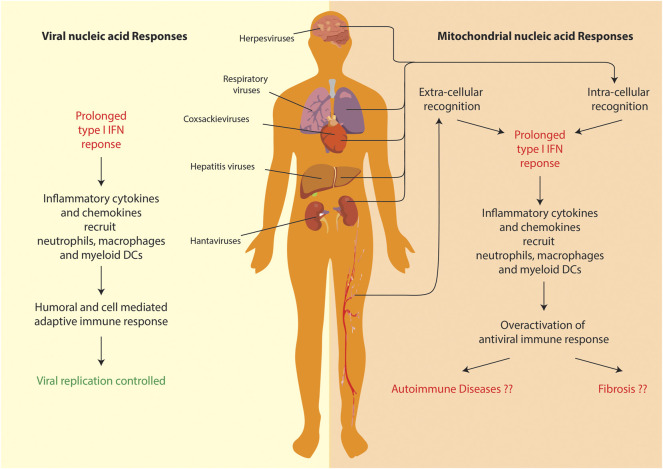
Comparison of anti-viral and mitochondrial nucleic acid responses. Both lead to a prolonged T1-IFN inflammatory response. However, in case of mitochondrial nucleic acid sensing pathways, the overactivation of inflammation leads to systemic/organ-specific auto-immune diseases or even fibrosis in various organs.

The field of mitochondrial nucleic acid-sensing has, in the recent years, made rapid progress owing to attention it drew from cell biologists, immunologists and clinicians alike. Since, the phenomenon directly connects all these branches of biology, the study requires a comprehensive understanding of all its aspects—including the nature of the molecular patterns, release and recognition in the cytoplasm, signaling and resulting patho-physiologies. Due to the bacterial ancestry of the mitochondrial genome, the molecular sensors of innate immunity can be easily repurposed to detect its extra-mitochondrial presence. Additionally, since an elaborate mechanism of anti-viral immunity exists to deal with viral pathogens, the molecular sensors involved can be also repurposed to detect mitochondrial nucleic acids outside their regular confines. However, it the response that mitochondrial nucleic acids garner from the over activation of antiviral immunity, which leads to a systemic patho-physiology (Figure 4). Additionally, a lot of critical shortcomings need to be addressed particularly in the areas of mitochondrial nucleic acids release and the etiology of pathologies. Let us look at them one by one.

The major models of release from the mitochondrial matrix involve either 1. herniation of the inner membrane through BAX/BAK mediated outer membrane pore (McArthur et al., 2018) or 2. mPTP mediated channel pore formation (Kim et al., 2019). Both models require thorough mechanistic examination. Herniation would not lead to a vesicle like structure to protrude out form the outer membrane. The issue of how this vesicle gets permeabilized to release the mitochondrial nucleic acids into the cytoplasm has never been addressed. The molecular pathway of apoptotic caspase inactivation for BAX/BAK to generate the pore also needs to worked out. On the other hand, what causes the growth in pore size of the mPTP so as to allow the passage of the bulky nucleoid needs to be investigated. Furthermore, what is the effect of such a huge pore on the mitochondrial structural integrity needs to be explored.

With more and more reports of increased occurrence of mitochondrial DNA in connection with multiple different diseases, it seems increasingly important to shed light on this aspect. The central question is whether mitochondrial DNA is a concomitant of the disease itself or is just a side effect. Also, it is import to determine if mtDNA sensing may have an additional secondary influence on the course of the disease, or whether it is a primary patho-mechanistic determinant of the progression of the disease itself. mtDNA copy numbers do influence the mitochondrial health and any dysfunctions in the maintenance process are associated with neuroinflammatory diseases. A concept was proposed in the study by Dölle et al. that, dysregulation of mtDNA homeostasis might be a key process in the pathogenesis of neuronal loss in Parkinson’s disease (Dölle et al., 2016). Physiologically, the amount of mtDNA in substantia nigra dopaminergic neurons increases with age, such that the wild-type mtDNA population is maintained in good numbers despite increasing deletions. This upregulation seems absent in PD patients, resulting in a depletion of the wild-type mtDNA population. In contrast, neuronal mtDNA point mutation load was not increased in PD, wich might be the trigger for its release and signaling. In a different context, the involvement of mtDNA signaling in pathogenesis of Alzheimer’s disease (AD) is also currently under discussion, as several reports of mitochondrial dysfunction and also the dysfunction of Pink1/Parkin, excessive ROS production are reported in association with AD (Witte et al., 2009; Ye et al., 2015). However, a direct contribution of mtDNA in pathogenesis of AD was not proven yet, the inflammatory component, showing induction of the inflammasome, cytokines and NF-ΚB might hint a correlation of mtDNA accumulation in AD (Heneka et al., 2013; Ahmed et al., 2017; Hu et al., 2021).

Apart from the biogenesis and maintenance of mitochondrial nucleic acids, mitochondrial quality control might also play a role in various diseases. In pulmonary disorders, PINK1 was reported to attenuate mtDNA release in alveolar epithelial cells (Bueno et al., 2019). Both, ER stress and PINK1 deficiency in AECII led to oxidation and damage of mtDNA and subsequent extracellular release, which was recognized by TLR9 after endocytosis. Besides the inflammatory response, mtDNA triggered secretion of the profibrotic factor TGF-β. In addition, mtDNA oxidation and damage were found in IPF human lungs and circulating mtDNA plasma- and bronchoalveolar lavage levels were significantly elevated in patients with idiopathic pulmonary fibrosis (IPF). Strikingly, the induction of inflammation and autophagy seems to be cross-talking via STING, as activation of STING interfered with lysosomal acidification, hence disturbing autophagy in mtDNA mediated sepsis-induced acute lung injury. As a consequence, the induction of autophagy or STING deficiency alleviated lung injury (Liu et al., 2021). The proper functioning of the mitophagy also seems to be of great importance for the preservation of renal function. On the one hand, mitophagy seems to be essential in ischemic renal situations (Tang et al., 2018; Livingston et al., 2019), on the other hand, it also appears to be indispensable in the prevention of inflammatory and fibrotic processes (Szeto et al., 2016; Bhatia and Choi, 2019). Further, the role of mitophagy and its function to remove mitochondrial material has to be discussed, since inhibition of mitophagy molecules caused TLR9-mediated inflammatory responses in cardiomyocytes, myocarditis and dilated cardiomyopathy (Oka et al., 2012). Mitophagy-mediated mtDNA release aggravates stretching-induced inflammation and lung epithelial cell injury via the TLR9/MyD88/NF-κB pathway (Jing R et al., 2020). Thus, the molecular connections with autophagy/mitophagy and mitochondrial nucleic acid sensing pathways need to be examined in detail.

Lastly, the nature of the trigger and site of nucleic acid sensing also varies a lot for different diseases. Thus, we briefly touch upon the distinction of intracellular and extracellular DNA sensing. Usually there are two possible situations which lead to the occurrence of circulating mtDNA. The first situation may be a severe (acute) destruction of tissue. The other would be, mitochondria-related dysfunctions and resulting (chronic) pathologies of the organ system. However, the distinction between the two can be often murky as cell free mtDNA from acute damage in tissue might also contribute to additional inflammatory reactions in the tissue itself. Clinically, this might present as if the organ system failed and thus skew the interpretation towards the second hypothesis. Thus, while looking at the etiology of such pathologies one needs to carefully dissect the origin of the insult or signal. Similarly, although sensing of mitochondrial DNA/RNA is a widely established trigger for an enhanced (interferon) immune response, there appear to be fundamental differences in different cell/organ types that are studied. This is underscored by Brachene et al., who showed that triggering an interferon response by increased mtRNA is, by no means, a universal process (Brachène et al., 2021). Rather, it seems to depend not only on specific organ systems but also on the proliferative status of the cells. Also, the nucleic acid sensing pathways could aggravate the triggers for other unassociated disorders. Using the example of the influence of mitochondrial dysfunction in the pathogenesis of Parkinson’s disease, it can be deduced that dysfunction in the mitochondrial system alone is not sufficient to trigger the disease. Much more, the chronic proinflammatory stress could be compensated in case of an intact regulatory system, which, however, could be omitted in case of another mutation affecting this very compensatory mechanism. Thus, similar to tumorigenesis, a “second hit” hypothesis is being attempted: In addition to an underlying dysfunction in the mitochondrial system itself, it could be a second malfunction in the inflammatory pathway regulating mechanisms that ultimately triggers the onset of mitochondrial genome-driven disease.

However, there are a few studies which clearly contradict the role of mitochondrial nucleic acid sensing pathways in diseases. Studies in the autoimmune disease rheumatoid arthritis, show T cells in RA patients when treated with mtDNA actually have lower IFNB and IFIT1 transcripts (Li Y. et al., 2019). In other studies of chronic heart failure, although those patients show significantly higher levels of mtDNA than age- and sex-matched healthy controls, there is no association between the severity of heart failure and the levels of serum mtDNA (Dhondup et al., 2016). Additionally, one study even found TLR9 is not strongly involved in mtDNA-induced inflammation caused by cardiac ischemic injury (Omiya et al., 2016). Based on experiments using TLR9 null mice, they showed no differences in the number of infiltrating inflammatory cells and the levels of inflammatory cytokine mRNA in infarct hearts between TLR9-deficient and wild-type mice. Thus TLR9, in opposition to its role in invoking inflammation, actually promoted proliferation and differentiation of cardiac fibroblasts for cardiac remodeling. Thus, it is very clear that a thorough examination of mitochondrial nucleic acid sensing pathways is necessary. But overwhelming evidence do point in a direction of benefits of the pathway in diagnostics and treatments.

To conclude, the new insights into the key players of the patho-mechanisms of autoimmune diseases also bring forward new therapeutic options for discussion. Currently, the blocking of cGAS-STING pathways but also TLR are intensively discussed as potential therapeutic targets for the treatment of autoimmune diseases, for example SLE (Decout et al., 2021; Fillatreau et al., 2021). Apart from autoimmune diseases, circulating mtDNA can affect organ systems as well. Mechanically-induced cartilage injury also leads to leak of mtDNA into the synovial fluid through cell death/rupture (Seewald et al., 2020). Again, treatments with mitoprotective SS31 peptide, which interacts specifically with CL to affect membrane curvature and prevent peroxidative damage, significantly lower circulating mtDNA bringing it to similar levels to that of the controls. This clearly indicates a great potential for developing or re-tasking drugs for treating these systemic inflammatory diseases associated with mitochondrial energetics and metabolism. Additionally, new biomarkers of the mitochondrial nucleic acid sensing pathways could accelerate timely detection and life-saving therapeutic interventions (Borsche et al., 2020; Ward et al., 2021). Hence, it is necessary to have a comprehensive overview of the mitochondrial nucleic acid sensing pathways in order to study diseases of the OXPHOS dysfunction.
